# Actions of Thiols, Persulfides, and Polysulfides as Free Radical Scavenging Antioxidants

**DOI:** 10.1089/ars.2022.0191

**Published:** 2023-10-16

**Authors:** Noriko Noguchi, Yoshiro Saito, Etsuo Niki

**Affiliations:** ^1^ The Systems Life Sciences Laboratory, Department of Medical Life Systems, Faculty of Life and Medical Sciences, Doshisha University, Kyotanabe, Japan.; ^2^ Laboratory of Molecular Biology and Metabolism, Graduate School of Pharmaceutical Sciences, Tohoku University, Sendai, Japan.; ^3^ Research Center for Advanced Science and Technology (RCAST), The University of Tokyo, Meguro-ku, Japan.

**Keywords:** antioxidant, free radical, lipid peroxidation, glutathione, hydrosulfides, persulfides, polysulfides, thiol

## Abstract

**Significance:**

The essential roles of thiol compounds as redox signaling mediators and protectors have been established. Recently, the roles of persulfides and polysulfides as mediators involved in numerous physiological processes have been revealed.

**Recent Advances:**

Recently, it became possible to detect and measure persulfides and polysulfides in human fluids and tissues and their physiological functions, including cellular signaling and protection against oxidative stress, have been reported, but the underlying mechanisms and dynamics remain elusive.

**Critical Issues:**

Physiological functions of thiol compounds have been studied, focusing primarily on two-electron redox reactions. In contrast, the contribution of one-electron redox mechanisms, that is, free radical-mediated oxidation and antioxidation, has received much less attention. Considering the important effects of free radical-mediated oxidation of biological molecules on pathophysiology, the antioxidant functions of thiol compounds as free radical scavengers are challenging issues.

**Future Directions:**

The antioxidant actions and dynamics of thiols, hydropersulfides, and hydropolysulfides as free radical scavenging antioxidants and their physiological significance remain to be established. *Antioxid. Redox Signal.* 39, 728–743.

## Introduction

We aerobic organisms are constantly exposed to oxidative stress in return for efficient energy production by using molecular oxygen. Over the long course of evolution, we achieved an efficient antioxidant network to protect ourselves from detrimental oxidative stress induced by reactive oxygen, nitrogen, and sulfur species (Olson, 2019; Sies, 2021). Enzymatic and nonenzymatic antioxidant systems have evolved in aerobic cells and tissues.

Superoxide dismutases (SODs), catalase, glutathione peroxidases (GPxs), glutathione S-transferase, thioredoxin, and the peroxiredoxin (Prx) families are important enzyme antioxidants. The term “antioxidant” means many things. Antioxidants are compounds that inhibit deleterious oxidative modification of biological molecules by sequestering redox-active metal ions, reducing hydrogen peroxide and hydroperoxides, scavenging reactive oxygen and related species, repairing oxidative damage, and inducing expression of antioxidant compounds and proteins (Halliwell, 2022; Niki et al., 1995).

The preventive roles of antioxidants against detrimental oxidation of biological molecules have been studied extensively (Niki, 2010), especially their effects against lipid peroxidation have recently received renewed attention in relation to ferroptosis (Farmer et al., 2022; Lange and Olzmann, 2022; Shah et al., 2019; Ursini and Maiorino, 2020).

The antioxidant efficacy depends on multiple factors, including the nature of oxidants and substrates (Niki, 2021; Niki, 2018). Therefore, it is important to specify the responsible oxidants when considering the antioxidant effects against modification of biological molecules or targets.

Thiol compounds containing a carbon-bonded sulfhydryl group (-SH) participate not only in cellular antioxidant defenses but also in redox signaling and regulation of biological processes (Toohey and Cooper, 2014; Trujillo et al., 2016; Ulrich and Jakob, 2019; Winterbourn and Hampton, 2008). Glutathione (GSH) is a tripeptide comprising three amino acids, that is, γ-glutamate, cysteine, and glycine, and exists in most mammalian tissues.

GSH is the most abundant low-molecular-weight thiol in animal cells—the intracellular concentrations ranging from 0.5 to 10 m*M*—affording protection against electrophiles (by conjugation and excretion) and hydrogen transfer or redox reactions against oxidative free radical damage (Sies, 1999). It has been established that GSH plays an important role in supporting the action of enzymes, including GPxs (Brigelius-Flohé and Flohé, 2020; Flohe, 2016).

GSH acts as a detoxifying agent that metabolizes harmful chemical species to harmless inactive products. GSH is also involved in the formation and maintenance of disulfide bonds in proteins and in the transport of amino acids across cell membranes (Koppula et al., 2021).

Thiol groups in protein cysteine residues are involved in regulation of protein activity and redox signaling as well as in detoxification of reactive species and oxidative damage processes (Lorenzen et al., 2020; Sen, 2001). Cysteine thiols and their oxidized disulfide counterparts are balanced to maintain redox homeostasis in various cellular compartments, protect organisms from oxidative and xenobiotic stressors, and partake actively in redox regulatory and signaling processes, in many instances *via* the reversible reaction of thiol proteins by the two-electron redox mechanism (Sen, 2001). Thiols also react with multiple electrophiles to produce various adducts (Shibata and Uchida, 2019).

In general, physiological reactions mediated by enzymes proceed in a regulated manner, in which thiol compounds such as GSH and the protein cysteine residue contribute to the maintenance of homeostasis by nonradical mechanisms. However, it is conceivable that thiols may also act as antioxidants by scavenging reactive oxygen species and as protectants by repairing protein and deoxyribonucleic acid (DNA) base damage through H atom donation and/or electron transfer (Cuevasanta et al., 2015).

The role of thiols as radical scavenging antioxidants has been controversial (Chauvin et al., 2017; Kalyanaraman, 1995; Moosmann and Hajieva, 2022; Pascoe and Reed, 1989; Trujillo et al., 2016; Winterbourn, 2015), partly because thiols are not particularly reactive toward oxygen radicals and the thiyl radicals (RS^•^s) produced by one-electron oxidation of thiols are not stable, but may act as oxidants rather than antioxidants (Everett and Wardman, 1995; Moosmann and Hajieva, 2022).

Free radicals have been implicated in deleterious modification of biological molecules and pathogenesis of various diseases (Sies, 2021). For example, it has been observed that both the absolute concentrations and molar ratios of oxidation products to parent substrates of specific products by free radical-mediated lipid peroxidation such as (*trans*, *trans*)-hydro(pero)xy-octadecadienoate [H(p)ODE] and/or *racemic* H(p)ODE increase with progress of atherosclerosis (Niki, 2018) and nonalcoholic fatty liver diseases (NAFLDs) (Feldstein et al., 2010; Zein et al., 2012).

Higher levels of isoprostanes, biomarkers for lipid peroxidation, have been observed in the patients across multiple diseases compared with healthy subjects (Morrow and Roberts, 1997). The formation of lipid-derived radicals in *in vitro* and *in vivo* systems has been confirmed (Matsuoka and Yamada, 2021). The formation and reaction pathways of free radicals *in vivo* are in general random and difficult to be regulated. Free radicals are assumed to be damaging species that cause deleterious effects, rather than regulated signaling mediators.

Since the discovery that hydrogen sulfide is an endogenously generated signaling mediator (Abe and Kimura, 1996), it is becoming evident that reactive sulfur species, including dihydrogen sulfide (HS_n_H), hydropersulfide (RSSH), and polysulfide (RSS_n_R, *n* > 1, R = hydrogen or alkyl), are produced endogenously and exert multiple physiological functions through two-electron oxidation pathways and mechanisms (Barayeu et al., 2023; Cuevasanta et al., 2022; Fukuto, 2022; Fukuto et al., 2018; Griffiths et al., 2023; Iciek et al., 2022; Ida et al., 2014; Kimura, 2021; Olson, 2019; Olson and Gao, 2019; Olson et al., 2023; Sawa et al., 2022; Zhang et al., 2021; and references cited therein).

Significant levels (50–100 μ*M*) of persulfides and polysulfides have been reported in mammalian cells, tissues, and plasma (Ida et al., 2014). However, the underlying chemical and biochemical mechanisms associated with their physiological actions remain elusive. Traditionally, the physiological effects of reactive sulfur species have been considered in terms of two-electron redox mechanisms, while much less attention has been paid to the antioxidant action of thiols by the one-electron redox mechanism.

The objective of the present article is to consider the potential involvement of free radical-mediated actions of the abovementioned multiple sulfides as antioxidants, especially against lipid peroxidation.

## Antioxidants and Antioxidant Capacity

The general issues of antioxidant effects on free radical-mediated oxidation are briefly considered. The physiological antioxidant effect is determined by multiple factors, including the concentration, localization, and mobility of the antioxidant at the reaction site; fate of the antioxidant-derived radical; and interaction with other antioxidants; as well as the inherent reactivity of the antioxidant toward free radicals.

The antioxidant (IH) needs to scavenge the free radical (X^•^) much faster and before the radical attacks the substrate (RH), that is, the rate of Reaction 1 should be much higher than that of Reaction 2:


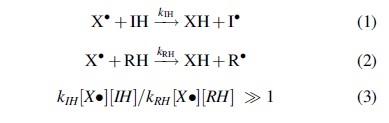


Importantly, multiple free radicals with different reactivities and selectivities are involved in oxidation of biological molecules *in vivo*, and Equation 3 implies that the antioxidant effect depends on reactivities of both attacking radicals and the substrates being oxidized and their concentrations.

The chemical properties and reactivities of GSH, cysteine, hydropersulfide, ascorbate, α-tocopherol, and linoleate, the most abundant polyunsaturated fatty acid (PUFA), toward biologically relevant free radicals and oxidants are summarized in Table 1.

**Table 1.** Chemical Properties and Reactivities of Glutathione, Cysteine, Hydropersulfide, Ascorbate, α-Tocopherol, and Linoleate Toward Biologically Relevant Free Radicals and Oxidants

	GSH	CySH	RSSH	Ascorbate	α-TOH	Linoleate
*D*(S,O,C-H)^a^	87 [1]	86 [1]	70 [2]	n.a.	77 [3]	75 [4]
Redox potent^b^	920 [5]	920 [6]	680 [6]	282 [6]	500 [6]	600 [7]
pKa	8.93 [8]	8.45 [8]	5.45 [9]	4.2 [10]	13.1 [11]	
Log P^c^	−6.4 [10]	−2.49 [10]	n.a.	−1.85 [10]	3.36 [10]	n.a.
Rate constant *k*^d^						
*R*^•^	10^7^ [1]	0.3–5 × 10^7^ [1]	5 × 10^8^ [12]	n.a.	n.a.	n.a.
RS^•^	6 × 10^7^ [13]	n.a.	1 × 10^10^ [12]	3 × 10^8^ [14]	n.a.	10^6^∼10^7^ [15]
HO^•^	1.3 × 10^10^ [16]	4.7 × 10^10^ [16]	n.a.	1 × 10^10^ [16]	∼10^10^ [17]	10^9^ [17]
RO^•^	6.62 × 10^7^ [1]	1.15 × 10^8^ [1]	1 × 10^9^ [12]	n.a.	∼ 10^9^ [17]	9 × 10^9^ [17]
RO_2_^•^	4.24 × 10^3^ [1]	1.07 × 10^4^ [1]	2 × 10^6^ [12]	2x10^6^ [17]	3 × 10^6^ [18]	62 [19]
HO_2_^•^	7.32 × 10^3^ [1]	1.81 × 10^4^ [1]	n.a.	n.a.	n.a.	1.2 × 10^3^ [17]
CO_3_^•−^	5.3 × 10^6^ [16]	1.6 × 10^7^ [16]	n.a.	1.1 × 10^9^ [16]	n.a.	>2 × 10^5^ [20]
NO_2_^•^	2 × 10^7^ [21]	5 × 10^7^ [21]	n.a.	3.5 × 10^7^ [16]	<10^6^ [16]	>2 × 10^5^ [20]
ONOOH	1.3 × 10^3^ [22]	5.9 × 10^3^ [23]	n.a.	n.a.	n.a.	n.a.
^1^O_2_	2.4 × 10^6^ [24]	8.3 × 10^6^ [24]	n.a.	1.9 × 10^6^ [25]	2.8 × 10^8^ [24]	10^5^ [26]
HOCl	1.1 × 10^8^ [27]	3.1 × 10^8^ [27]	n.a.	n.a.	n.a.	18 [28]
H_2_O_2_	0.87 [29]	0.84 [30]	n.a.	n.a.	n.a.	n.a.

Numbers in brackets show references: [1] Denisov et al. (2009); [2] Benson (1978); [3] Mulder et al. (2005); [4] Pratt et al. (2003); [5] Madej and Wardman (2007); [6] Buettner (1993); [7] Koppenol (1990); [8] Winterbourn and Hampton (2008); [9] Benchoam et al. (2020); [10] Pub Chem; [11] Mukai (2007); [12] Chauvin et al. (2017); [13] Nauser et al. (2004); [14] Chatgilialoglu et al. (2014); [15] Chatgilialoglu (1990); [16] Pryor et al. (2006); [17] Simic et al. (1992); [18] Burton et al. (1985); [19] Howard and Ingold (1967); [20] Everett et al. (1996); [21] Ford et al. (2002); [22] Trujillo and Radi (2002); [23] Radi et al. (1991); [24] Di Mascio et al. (1991); [25] Mukai et al. (2012); [26] Doleiden et al. (1974); [27] Storkey et al. (2015); [28] Everett et al. (1996); [29] Winterbourn and Metodiewa (1999); and [30] Portillo-Ledesma et al. (2014). The data were obtained at ambient temperature in solution.

^a^ Bond dissociation energy obtained by calculation for S-H bonds of GSH, cysteine, and hydropersulfide; phenolic O-H bond of α-tocopherol; and bisallylic C-H bond of linoleate in kcal/mol.

^b^ One-electron redox potential in mV.

^c^ Partition coefficient.

^d^ Rate constant *k* for reactions of free radicals and nonradical oxidants with GSH, CySH, RSSH, ascorbate, α-TOH, and linoleate in *M*^−1^·s^−1^.

α-TOH, α-tocopherol; CySH, cysteine; GSH, glutathione; n.a., not available; RS^•^, thiyl radical; RSSH, hydropersulfide.

Furthermore, the rate constants (*k*) for scavenging of multiple biological oxidants by thiols, hydropersulfides, ascorbate, and α-tocopherol, some being obtained experimentally, while others by calculation, are included in Table 1 (Buettner, 1993; Carballal et al., 2014; Davies, 2016; Denisov et al., 2009; Madej and Wardman, 2007; Wardman, 1989). It is noted that the data shown in Table 1 depend on several conditions, including temperature, solvent, and pH, and accordingly the reported values do not always match.

The pKa values of GSH, GSSH, and ascorbic acid are reported as 8.93, 5.45, and 4.2, respectively (Table 1), indicating that under physiological conditions, GSH is predominantly protonated, while GSSH and ascorbic acid exist as monoanions.

Ascorbate scavenges radicals by electron transfer, while GSH and α-tocopherol react with radicals by hydrogen atom donation. The rate constants for the reaction of GSH and cysteine with the secondary alkyl peroxyl radical were estimated at 4.24 × 10^3^ and 1.07 × 10^4^ *M*^−1^·s^−1^ at 37°C in a nonpolar solution (Denisov et al., 2009). Table 1 also shows that RSSH scavenges alkyl, alkoxyl, and peroxyl radicals faster than GSH.

Equation 3 suggests that for the antioxidant to act efficiently, the *k*_IH_[IH]/*k*_RH_[RH] ratio should be at least larger than 10. Since the molar ratio of antioxidant to substrate ([IH]/[RH]) *in vivo* is in general less than 1/100, the *k*_ith_/*k*_RH_ ratio should be larger than 10^3^ (Niki, 2018). Only a few absolute rate constants have been reported for the reactions of peroxyl radicals with thiol compounds.

The rate constant for scavenging peroxyl radicals by GSH is around 10^3^ *M*^−1^·s^−1^ (Chauvin et al., 2017), only about an order larger than the rate constants for chain propagation (*k*p) in peroxidation of linoleate and arachidonate, *k*p = 62 *M*^−1^·s^−1^ (Howard and Ingold, 1967) and 197 *M*^−1^·s^−1^ (Xu et al., 2009), respectively.

The rate constants in Table 1 indicate that when linoleate is considered as a substrate, the *k*_GSH_/*k*_linoleate_ ratios for thiyl, hydroxyl, alkoxyl, alkyl peroxyl, hydroperoxyl, carbonate anion, and nitrogen dioxide radicals are all less than 10^3^, indicating that GSH is not an efficient radical scavenging antioxidant against lipid peroxidation.

On the other hand, α-tocopherol and ascorbate (the major lipophilic and hydrophilic antioxidants, respectively, in humans) scavenge peroxyl radicals with the rate constants around 10^6^ *M*^−1^·s^−1^, suggesting that α-tocopherol and ascorbate are roughly 1000 times more efficient than GSH for scavenging lipid peroxyl radicals.

Of note, the *k*_GSH_/*k*_linoleate_ ratio for hypochlorite (HOCl) is as high as 10^7^. It was shown that low-molecular-weight thiols such as GSH and sulfur-containing amino acids in proteins were major targets for HOCl, the rate constant being higher than 10^8^ *M*^−1^·s^−1^ (Davies, 2016; Storkey et al., 2014).

Thiols (RSH) react rapidly with HOCl to yield sulfenyl chlorides (RSCl), which decompose to give thiyl radicals (Davies and Hawkins, 2000). GSH may repair protein damage by donating hydrogen to the protein radical. However, it was estimated that under physiological conditions, such effects of GSH were much less than that of ascorbate (Gebicki et al., 2010).

Interaction between antioxidants is another factor that determines the antioxidant effect. For example, α-tocopherol and ascorbate inhibit lipid peroxidation synergistically, that is, α-tocopherol scavenges the lipid peroxyl radical in the lipophilic domain where lipid peroxidation proceeds and the resulting α-tocopheroxyl radical is reduced by ascorbate to regenerate α-tocopherol and inhibit tocopherol-mediated peroxidation (Bowry et al., 1992; Niki et al., 1995).

In the presence of ascorbate, α-tocopherol is spared, but when ascorbate is completely depleted, α-tocopherol consumption and accumulation of lipid hydroperoxides begin. It becomes more difficult for ascorbate to scavenge radicals as the radicals go deeper into the interior of membranes and lipoproteins (Gotoh et al., 1996; Takahashi et al., 1988).

GSH and cysteine are also capable of reducing the α-tocopheroxyl radical, and rate constants decrease in the order of ascorbate > cysteine > GSH (Motoyama et al., 1989; Niki et al., 1982; Tsuchihashi et al., 1995). Even though the rate constant for reduction of the α-tocopheroxyl radical by GSH is smaller than that by ascorbate, when one considers high concentration of GSH in cytosol, 1–10 m*M* (Beutler and Gelbart, 1985; Jones et al., 2000), the reduction by GSH may not be ruled out in cells.

It was reported that both enzymatic and nonenzymatic lipid peroxidation processes of the rat liver microsomal system were inhibited in the presence of both GSH and vitamin E, which was ascribed to the role of GSH in maintaining vitamin E in the reduced state (Scholz et al., 1989). On the other hand, Barclay (1988) concluded that GSH did not act synergistically by regenerating α-tocopherol from the tocopheroxyl radical in liposomal membranes. It was reported that hydrogen sulfide reduced quinone to hydroquinone, which may serve as a free radical scavenging antioxidant (Olson et al., 2022).

It should be noted that rate constants for scavenging oxidizing species are dependent on the solvent. The hydrogen bonding interaction between the antioxidant and solvent slows scavenging of radicals by the antioxidant (Chauvin et al., 2017); for example, the rate constant for scavenging the peroxyl radical by α-tocopherol is 3.2 × 10^6^ *M*^−1^·s^−1^ in chlorobenzene (Burton and Ingold, 1984), but is diminished to 5.1 × 10^5^ *M*^−1^·s^−1^ in *tert*-butyl alcohol (Niki et al., 1984).

Another factor that affects antioxidant action is localization of antioxidants in the medium. Hydrophilic uric acid, which is less reactive than ascorbate, suppresses consumption of α-tocopherol during oxidation of low-density lipoprotein (LDL) in the aqueous dispersion induced by peroxyl radicals produced in the aqueous phase, but uric acid cannot spare α-tocopherol when peroxyl radicals are produced within LDL particles (Sato et al., 1990). Uric acid, unlike ascorbate, does not reduce the α-tocopheroxyl radical to regenerate α-tocopherol, but can scavenge peroxyl radicals in the aqueous phase faster than α-tocopherol, which exists within LDL particles.

This shows that antioxidant efficacy is determined by localization of antioxidants, radicals, and substrates as well as the reactivity of the antioxidant. GSH concentrations in normal human plasma and cytosol are several micromolar and millimolar, respectively (Beutler and Gelbart, 1985; Jones et al., 2000) and hence GSH may exert substantial antioxidant effects against cellular lipid peroxidation. It may be noteworthy that circulating plasma levels of total GSH decreased with age, the downward trend being monotonic from about 4 μ*M* (45–55 years) to 2 μ*M* (>75 years) (Malaeb et al., 2022).

Another example of the localization effect is the competition between α-tocopherol and β-carotene for scavenging peroxyl radicals in the membranes and lipoproteins. α-Tocopherol and β-carotene exist at the surface and interior of the membrane, respectively. α-Tocopherol, which is about an order of magnitude more reactive than β-carotene toward peroxyl radicals, scavenges aqueous peroxyl radicals faster and spares β-carotene efficiently, but β-carotene scavenges peroxyl radicals localized within the membrane faster than α-tocopherol (Tsuchihashi et al., 1995).

## Thiyl Radicals: Formation, Chemical Properties, and Reactions

The thiyl radical (RS^•^) is produced when thiols scavenge free radicals by hydrogen atom donation or by proton donation, followed by electron release from the thiolate anion (Asmus, 1990; Schöneich, 2016). The two processes are stoichiometrically equivalent and may be difficult to distinguish experimentally because proton transfer reactions to solvent water are usually fast. The one-electron reduction of disulfides also produces thiyl radicals *via* an intermediate disulfide radical anion (Reaction 4):

(4) $$RSSR+e^{-}\to RSSR^{∙-}\to RS^{∙}+RS^{-}$$

Bonini and Augusto (2001) characterized thiyl, sulfinyl RSO^•^, and disulfide anion RSSR^•−^ radicals produced from both GSH and cysteine by the reaction with peroxynitrite. The thiyl radical may also be generated by photolysis of RSNO and the reaction of thiol compounds with HOCl *via* decomposition of sulfenyl chlorides (RSCl) (Davies and Hawkins, 2000).

Thiyl radicals have been detected by spin trapping in several studies (Davies and Hawkins, 2000; Harman et al., 1984; Niki et al., 1982; Stoyanovsky et al., 2011). The reaction of alkoxyl radical with GSH under vacuum in the presence of a spin trap α-(4-pyridyl-1-oxide)-*N*-*tert*-butylnitrone (POBN) gave a doublet of triplets electron spin resonance (ESR) spectrum, the coupling constant being *a*^N^ = 1.523 and a_β_^H^ = 0.228 mT, which was ascribed to a spin adduct of GSH thiyl radical (Niki et al., 1982).

Interestingly, when GSH was added to a solution containing the α-tocopheroxyl radical and POBN, the ESR spectrum of the α-tocopheroxyl radical was replaced by the ESR spectrum of the GSH thiyl radical spin adduct, suggesting that GSH reduced the α-tocopheroxyl radical to give α-tocopherol and the GSH thiyl radical. Similarly, the reaction of cysteine and the α-tocopheroxyl radical gave a spin adduct of the cysteine thiyl radical and POBN, the hyperfine splitting constants being a_N_ = 1.504 and a_β_^H^ = 0.230 mT (Motoyama et al., 1989; Tsuchiya et al., 1985). The reaction rates with the galvinoxyl radical decreased in the order of ascorbate, cysteine, and GSH in acetone/water solution, micelle, and liposomal membranes (Tsuchiya et al., 1985).

Different spin traps have been developed and used for the analysis of thiyl radicals. It was shown that thiyl radicals were trapped with a spin trap 5,5-dimethyl-1-pyrroline-N-oxide (DMPO) giving characteristic spin adducts with hyperfine splitting constants, a^N^ = 1.52–1.58, a^H^ = 1.52–1.80 mT, and g values in the range 2.0065–2.0067 for DMPO adducts (Davies et al., 1987). It was reported that N-*tert*-butyl(methylideneamine) N-oxide reacted with the GSH radical to form a spin adduct, which exhibited a distinct ESR spectrum (Kondo et al., 1997).

Furthermore, it was reported that the mitochondria-targeted 5-diethoxyphosphoryl-5-methyl-1-pyrroline N-oxide (Mito-DEPMPO)-SG spin adduct, formed by trapping of the glutathiyl radical by Mito-DEPMPO, was persistent and ESR parameters were distinctly different and highly characteristic (Hardy et al., 2007).

### Fate of thiyl radicals

The fate of thiyl radicals formed concomitantly when thiol compounds scavenge radicals determines the antioxidant effects of thiol compounds. Thiyl radicals may undergo multiple secondary reactions, either inter- or intramolecularly, including hydrogen abstraction, electron transfer, addition to the double bond, recombination, and rearrangement. Compared with the phenoxyl radicals derived from phenolic antioxidants, thiyl radicals undergo diverse reactions with oxygen and biological molecules, which complicates the assessment of antioxidant effects of thiol compounds.

Kinetic studies have been performed and the absolute rate constants for reactions of thiyl radicals with oxygen and biological molecules have been measured, as summarized in Table 2 (Asmus, 1990; Chauvin et al., 2016; D'Aquino et al., 1989; Denisov et al., 2009; Kaneko et al., 2022; Nauser et al., 2015; Schöneich et al., 1989; Wardman and von Sonntag, 1995).

**Table 2.** Rate Constants for Reactions of Thiyl GS^•^, Perthiyl RSS^•^, and Peroxyl Radicals with Oxygen, Sulfides, Lipids, and Antioxidants

Radical	GS^•^	Perthiyl RSS^•^	Peroxyl RO_2_^•^
Substrate			
O_2_ *k*_1_	2 × 10^9^ [1]	Slow [16]	n.a.
*k_−_*_1,_ s^−1^	6.2 × 10^5^ [1]		
NO	2 ∼ 3 × 10^9^ [2]	Slow [16]	n.a.
HSSH	n.a.	n.a.	3.5 × 10^5^ [15]
HSSSH	n.a.	n.a.	4.0 × 10^5^ [15]
HSSSSH	n.a.	n.a.	6.0 × 10^5^ [15]
RSSR *k*_1_	3.8 × 10^6^ [3]	n.a.	n.a.
*k_−_*_1,_ s^−1^	2.3 × 10^4^ [3]	n.a.	n.a.
Methyl oleate 18:1			
Addition	1.6 × 10^5^ [6,17]	n.a.	n.a.
Elimination (s^−1^)	1.7 × 10^7^ [6,17]		
Linoleate 18:2	1 × 10^7^ [4,5]	n.a.	62 [6]
Arachidonate 20:4	2 × 10^7^ [4,5]	n.a.	197 [7]
Addition *k*_1_	1.6 × 10^5^ [8]		
Elimination *k_−_*_1,_ s^−1^	1.7 × 10^7^ [8]		
Peptides	10^3^ ∼ 10^5^ [9]	n.a.	n.a.
Ascorbate	3.6 × 10^8^ [5]	1 ∼ 6 × 10^6^ [10]	2.2 × 10^6^ [11]
α-Tocopherol	1 × 10^8^ [5]	n.a.	3.2 × 10^6^ [12]
Retinol	1.4 × 10^9^ [5]	n.a.	n.a.
β-Carotene	2.2 × 10^8^ [13]	n.a.	10^5^ [14]

The rate constants are shown in *M*^−1^·s^−1^ except for the first-order reaction, which is in s^−1^. Numbers in brackets show references: [1] Tamba et al. (1986); [2] Madej et al. (2008); [3] Symons (1974); [4] Schöneich et al. (1992); [5] D'Aquino et al. (1989); [6] Howard and Ingold (1967); [7] Xu et al. (2009); [8] Tartaro Bujak et al. (2016b); [9] Nauser and Schöneich (2003); [10] Everett et al. (1992); [11] Packer et al. (1980); [12] Burton et al. (1985); [13] Everett et al. (1996); [14] Tsuchihashi et al. (1995); [15] Kaneko et al. (2022); [16] Chauvin et al. (2016); and [17] Chatgilialoglu et al. (2005).

**FIG. 1.**
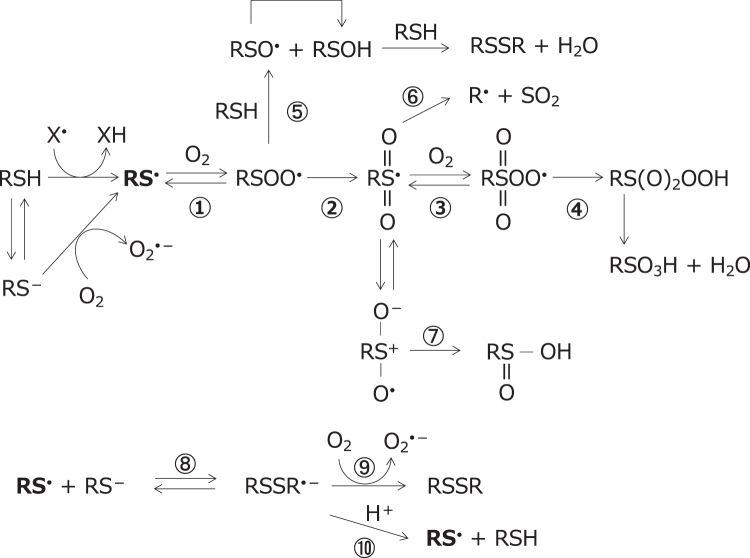
**Reaction of the thiyl radical, RS^•^, with molecular oxygen.** The thiyl radical reacts reversibly with molecular oxygen to yield the thiyl peroxyl radical, RSOO^•^ (Reaction 1). The thiyl peroxyl radical, RSOO^•^, rearranges to the sulfonyl radical, RS(O_2_)^•^ (Reaction 2), which subsequently reacts with molecular oxygen to form the sulfonyl peroxyl radical, RS(O_2_)OO^•^ (Equation 3), and ultimately yields sulfonates (Reaction 4). The thiyl peroxyl radical reacts with thiol to give the sulfinyl radical and sulfenic acid, RSOH (Reaction 5). The sulfonyl radical dissociates into the alkyl radical and SO_2_ (Reaction 6) or rearranges to give sulfinic acid, RS(O)OH (Reaction 7). In the presence of excess thiolate RS^−^, the thiyl radical exists in equilibrium with thiolate as a disulfide radical anion, RSSR^•−^ (Reaction 8), which may yield superoxide (Reaction 9) or thiyl radical (Reaction 10). Thiyl radicals may also react with lipids, proteins, and DNA bases to induce free radical-mediated oxidation. DNA, deoxyribonucleic acid; RS^•^, thiyl radical.

The reaction of thiyl radicals with molecular oxygen has been the subject of extensive studies and arguments (Bianco et al., 2016; Chauvin et al., 2017; Mönig et al., 1987). Thiyl radicals react with oxygen to give a variety of products by multiple routes, as outlined in Figure 1 (Chauvin et al., 2017; Iciek et al., 2022; Schöneich, 2019; Schöneich, 2016; Sevilla et al., 1990; Sevilla et al., 1988).

Thiyl radicals react reversibly with molecular oxygen to yield the thiyl peroxyl radical, RSOO^•^, the rate constants for forward and reverse reactions being reported as 2.2 × 10^9^ *M*^−1^·s^−1^ and 6.2 × 10^5^ s^−1^, respectively (Zhang et al., 1994). The thiyl peroxyl radical, RSOO^•^, rearranges to the sulfonyl radical, RS(O_2_)^•^, which subsequently reacts with molecular oxygen to form the sulfonyl peroxyl radical, RS(O_2_)OO^•^. In the presence of an electron or hydrogen donor, sulfonyl radicals convert into sulfonates, while sulfonyl peroxyl radicals ultimately yield sulfonates (Sevilla et al., 1988). The reported rate constants for the reaction of the thiyl radical and oxygen molecule vary considerably.

Thiyl radicals may also react with lipids, proteins, and DNA bases to induce free radical-mediated oxidation. The involvement and action of thiyl radicals in protein modification and degradation have been reviewed by Schöneich (2016). Protein thiyl radicals attack protein C-H bonds *via* intramolecular hydrogen transfer, which may lead to secondary amino acid oxidation and irreversible protein aggregation and/or fragmentation (Nauser et al., 2004).

The reported rate constants for hydrogen atom abstraction by the thiyl radical from peptides ranged from 1.7 × 10^3^ to 4 × 10^5^ *M*^−1^·s^−1^ (Nauser and Schöneich, 2003), from carbohydrates ranged from 1 to 3 × 10^4^ *M*^−1^·s^−1^ (Pogocki and Schöneich, 2001), and from amino acid side chain C-H bonds ranged between 1 × 10^3^ *M*^−1^·s^−1^ (Val) and 1.6 × 10^5^ (Ser) *M*^−1^·s^−1^ (Nauser et al., 2004). The thiol group of cysteine (Cys) is a predominant target for oxidative modification, where one-electron oxidation leads to formation of the cysteine thiyl radical, CysS^•^.

Thiyl radicals attack lipids to induce lipid peroxidation. The rate constants of the reactions of thiyl radicals with PUFA have been reported by several groups (D'Aquino et al., 1989; Doleiden et al., 1974; Schöneich et al., 1992; Schöneich et al., 1989). For example, rate constants for reactions of the GSH thiyl radical with arachidonate, linolenate, and linoleate were reported as 2.2 × 10^7^, 1.9 × 10^7^, and 1.3 × 10^7^ *M*^−1^·s^−1^, respectively, in methanol/water (D'Aquino et al., 1989). They are typically of the order of 10^7^ *M*^−1^·s^−1^, which is much larger than the rate constants for reactions of the peroxyl radical with PUFA, about 10^2^ *M*^−1^·s^−1^, suggesting that (as mentioned above) scavenging of peroxyl radicals by thiol compounds does not result in chain breaking, but rather chain transfer (Kunath et al., 2020).

Thiyl peroxyl radicals were found to be rather unreactive toward PUFAs, in contrast to isomer sulfonyl radicals. On the other hand, the rate constant for hydrogen atom abstraction from methyl linoleate by the α-tocopheroxyl radical was reported as 2.7 × 10^−2^ *M*^−1^·s^−1^ at 37°C in ethanol (Watanabe et al., 2000), showing that α-tocopherol has ideal characteristics of a chain-breaking antioxidant. Thiyl radicals derived from GSH, cysteine, and penicillamine react with nitric oxide through the S-nitrosation process, the rate constants being 2–3 × 10^9^ *M*^−1^·s^−1^ (Madej et al., 2008).

Furthermore, thiyl radicals add to the double bond of unsaturated fatty acids (reversibly) to convert *cis*-fatty acids into *trans*-fatty acids in a catalytic cycle (Chatgilialoglu et al., 2017; Chatgilialoglu et al., 2014; Ferreri et al., 2005). Thiyl radicals abstract bis-allylic hydrogen from PUFA faster than addition to double bonds, but thiyl radicals react with oleic acid, which lacks bis-allylic hydrogen, predominantly by addition to the double bond, and the reverse reaction results in *cis*–*trans* isomerization, giving rise to elaidic acid.

It was found that irradiation of linoleic acid in a homogeneous solution using ^60^Co source in the absence of thiol compounds gave linoleic acid hydroperoxides and that addition of 2-mercaptoethanol reduced the level of hydroperoxides, but induced *cis*–*trans* isomerization, showing dual effects of thiol compounds (Tartaro Bujak et al., 2016a). The effects of antioxidants against isomerization mediated by thiol compounds have been studied and inhibition of the isomerization process increased in the following order: α-tocopherol < ascorbate < all-trans retinol acetate (Chatgilialoglu et al., 2002; Tartaro Bujak et al., 2016a).

The sulfonyl radical, RS(O_2_)^•^, reacts with β-carotene by both electron transfer (Reaction 5) and addition to the double bond (Reaction 6) in an ∼3:1 ratio (Everett et al., 1996). The resulting β-carotene radical cation and adduct radicals are highly resonance stabilized and undergo slow bimolecular decay to nonradical products, but the β-carotene peroxyl radical may induce free radical oxidation of biological molecules.

These carotenoid-derived radicals react differently with oxygen, a factor that is expected to influence the antioxidant activity of β-carotene within tissues of varying oxygen tension *in vivo* (Burton and Ingold, 1984; Everett et al., 1996; Tsuchihashi et al., 1995).

(5) $$RS{\left(O_{2}\right)}^{∙}+\beta -carotene\to RS{\left(O_{2}\right)}^{-}+\beta -carotene^{∙+}$$

(6) $$RS{\left(O_{2}\right)}^{∙}+\beta -carotene\to {\left[RS\left(O_{2}\right)-\beta -carotene\right]}^{∙}$$

### Antioxidation

It must clearly be recognized that thiyl radicals are able to undergo a variety of reactions depending on the environmental conditions, including local concentrations of the reactive entities. Thus, the antioxidant effects of thiol compounds are determined by multiple factors. Compared with vitamins C and E, scavenging of radicals by thiol compounds is slower and the resulting thiyl radicals are less stable, implying less efficient antioxidant capacity.

Nonetheless, thiol compounds have often been observed to inhibit oxidative damage induced by free radicals in *ex vivo* and *in vivo* models, although it is difficult to prove if the antioxidant effect is due to scavenging free radicals or by other mechanisms. A few cases are discussed below.

It was found that various oxidants induce erythrocyte hemolysis, which was inhibited by antioxidants. AAPH (2,2′-azobis(2-amidinopropane) dihydrochloride), a free radical-generating initiator, induced hemolysis, and the extent of hemolysis was directly proportional to the total amounts of free radicals formed independent of initial AAPH concentrations (Miki et al., 1987). Peroxynitrite also induced hemolysis, which was suppressed more efficiently by GSH than trolox and uric acid (Kondo et al., 1997). It may be noted that GSH exerted higher reactivity toward peroxynitrite than α-tocopherol and uric acid (Morita et al., 2019).

NAFLD, a hepatic manifestation of metabolic syndrome, is now the most common liver disorder affecting a high proportion of the population worldwide, and lipid peroxidation has been implicated in its pathogenesis (Sumida et al., 2013). It was found that AAPH administered intraperitoneally to mice caused damage to biological tissues, including fatty degeneration of the liver, which was suppressed by GSH, cysteine, and trolox (Terao and Niki, 1986).

It was further found that azo compounds given to mice either by intraperitoneal administration or by dissolving in drinking water induced triacylglycerol (TG) increase and concomitant phospholipid decrease in the liver (Sumida et al., 2013). Furthermore, lipid peroxidation products such as H(p)ODEs and hydro(pero)xyeicosatetraenoates (H(p)ETEs) were increased in the liver in association with the increase in TG.

Importantly, *trans*, *trans*-H(p)ODEs and H(p)ETEs, specific biomarkers of free radical-mediated lipid peroxidation, were increased in the liver. It is noteworthy that a pattern similar to that induced by high-fat diet was observed. These results indicate that free radicals as well as high-fat diet induce a fatty liver by similar mechanisms, in which lipid peroxidation may be involved. These findings suggest that GSH and other thiols suppress free radical-induced oxidative damage *in vivo*, although it does not prove that this was due solely to scavenging free radicals or by enhancing GPx4 activity.

α-Lipoic acid, also known as thioctic acid, is a naturally occurring organosulfur compound that is synthesized by plants and animals, including humans. Dihydrolipoic acid, the reduced form of lipoic acid, acts as a potent antioxidant by radical scavenging and also by reducing oxidized antioxidants, including vitamin C, vitamin E, and ubiquinol, a reduced form of coenzyme Q (Kagan et al., 1992; Packer et al., 1995). It has been reported by Packer et al. that lipoic acid protects the neuronal system from oxidative damage *in vivo* (Cui et al., 2006).

## Persulfides and Polysulfides

It is becoming evident that reactive sulfur species, including hydrogen persulfide, hydropersulfides, and polysulfides, are present in mammalian cells and tissues to exert important physiological functions, including redox modulatory effects and protective roles against oxidative damage (Cuevasanta et al., 2022; Fukuto, 2022; Iciek et al., 2022; Olson et al., 2023; Sawa et al., 2022; and references cited therein). In this article, hydropersulfide refers to both the protonated RSSH and deprotonated RSS^−^ forms.

The term “antioxidants” means many things, but the antioxidant action by these sulfides has not been specified. Interestingly, and somewhat strangely, the role of hydropersulfides and polysulfides as protectants against oxidative damage has been studied primarily in terms of two-electron redox mechanisms, while the contribution of one-electron oxidation, that is, the free radical mechanism, has received much less attention despite the fact that roles of hydropersulfides, hydropolysulfides, and dialkyl polysulfides as radical scavenging antioxidants have been brought to attention recently (Barayeu et al., 2023; Fukuto, 2022; Poon and Pratt, 2018).

In the 1990s, the antioxidant effects of persulfides on free radical-mediated oxidation were studied using pulse radiolysis (Everett and Wardman, 1995; Everett et al., 1996; Everett et al., 1994; Everett et al., 1992). It was reported that the free radical scavenging reactions of persulfides were qualitatively similar to, but quantitatively different from, those of the corresponding thiol antioxidants (Everett and Wardman, 1995). Compared with thiol counterparts, bond energies of the S-H bond in hydropersulfides are weaker by about 20 kcal/mol, perthiols are stronger acids, and further perthiyl radicals are less reactive.

The pKa values shown in Table 1 suggest that at physiological pH, a significant proportion of persulfides exist as the deprotonated perthiolate anion (RSS^−^), while many thiols are predominantly in the protonated form. These imply that hydropersulfides are more potent radical scavenging antioxidants than the corresponding thiol compounds. The enhanced reactivity of hydropersulfide (RSSH) and stability of the perthiyl radical, RSS^•^, are due to resonance stabilization of the unpaired electron on persulfide, which can be delocalized onto both sulfur atoms, an effect not available to the unpaired electron on sulfide species, RSH.

In fact, potent antioxidant actions of hydropersulfides and hydropolysulfides have been revealed in this decade and attracted much attention, especially in connection with ferroptosis (Álvarez et al., 2017; Amorati et al., 2012; Barayeu et al., 2023; Chauvin et al., 2017; Fukuto et al., 2018; Millikin et al., 2016; Nagai et al., 2021; Ono et al., 2014; Poon and Pratt, 2018; Saund et al., 2015; Wu et al., 2022).

Pratt and his colleagues have performed comprehensive studies on the antioxidant action of hydropersulfides (Chauvin et al., 2017; Poon and Pratt, 2018), polysulfides (Chauvin et al., 2019), sulfinic acid (Griesser et al., 2018), polysulfide oxide (Chauvin et al., 2016), and thiosulfinate (Li et al., 2015) and elegantly shown that these sulfides acted as potent radical scavenging antioxidants against free radical-mediated oxidation of organic compounds. It was reported that hydropersulfides are excellent inhibitors of tetrahydrofuran (THF) oxidation initiated by the azo initiator in chlorobenzene at 37°C (Chauvin et al., 2017).

The observed inhibition periods corresponded to stoichiometries of 1, suggesting that the perthiyl radicals (RSS^•^) produced following H atom transfer to peroxyl radicals do not contribute to either chain-propagating or chain-breaking events. Indeed, perthiyl radicals combined to give tetrasulfides (RSSSSRs) with a rate constant of 6 × 10^9^ *M*^−1^·s^−1^ (Chauvin et al., 2017). It was found that perthiyl radicals were stable and did not react with oxygen or NO (Bianco et al., 2016; Chauvin et al., 2019).

In support of the above notion, the data summarized in Table 1 show that both the bond dissociation energy of hydropersulfide D(RSS-H) and pKa values are smaller compared with GSH: 70 *versus* 87 kcal/mol and 5.4 *versus* 8.9, respectively, suggesting that hydropersulfides are better donors of hydrogen atoms and electrons than GSH. Of note, abstraction of bis-allylic hydrogen from linoleate by the perthiyl radical is endothermic by 5 kcal/mol. As expected, hydropersulfides and hydropolysulfides act as potent radical scavenging antioxidants, as shown below.

The rate constants for scavenging peroxyl radicals by cumyl-hydropersulfide (cumyl-SSH) and dodecyl-hydropersulfide measured for oxidation of THF in chlorobenzene at 37°C were reported as 8.0 × 10^5^ and 8.3 × 10^5^ *M*^−1^·s^−1^, respectively, which were similar to the rate constant for α-tocopherol, 7.1 × 10^5^ *M*^−1^·s^−1^, under the same conditions (Chauvin et al., 2017). Recently, the rate constant for scavenging peroxyl radicals by cumyl-SSH, tert-dodecyl-SSH, and benzyl-SSH was determined as 2 × 10^5^ *M*^−1^·s^−1^ for oxidation of egg phosphatidylcholine liposomal membranes in PBS at pH 7.4 and 37°C (Wu et al., 2022).

It was reported that dialkyl and diallyl polysulfides contained in vegetables act as antioxidants against lipid oxidation. For example, diallyl polysulfides and dimethyl polysulfides contained in garlic and cabbage suppressed *in vitro* and *in vivo* lipid oxidation (Higuchi et al., 2003; Horie et al., 1992; Horie et al., 1989), but neither underlying mechanisms nor quantitative dynamics were studied in detail.

Chauvin et al. (2019) studied the radical scavenging activity of dialkyl polysulfides, RS_n_R (*n* = 1–4), and the corresponding 1-oxides at 37°C, 100°C, and 160°C. They also assessed the capacity of dialkyl sulfides to inhibit oxidation of 1-hexadecane at 100°C. Of polysulfides, only the tetrasulfide inhibited PBD-BODIPY oxidation, but sulfide, disulfide, and trisulfide did not inhibit oxidation.

The rate constant for scavenging the peroxyl radical by di-*tert*-butyl tetrasulfide was obtained as 2.3 × 10^5^ *M*^−1^·s^−1^ at 100°C. However, none of the polysulfides were able to inhibit oxidation at 37°C (Chauvin et al., 2019).

**FIG. 2.**
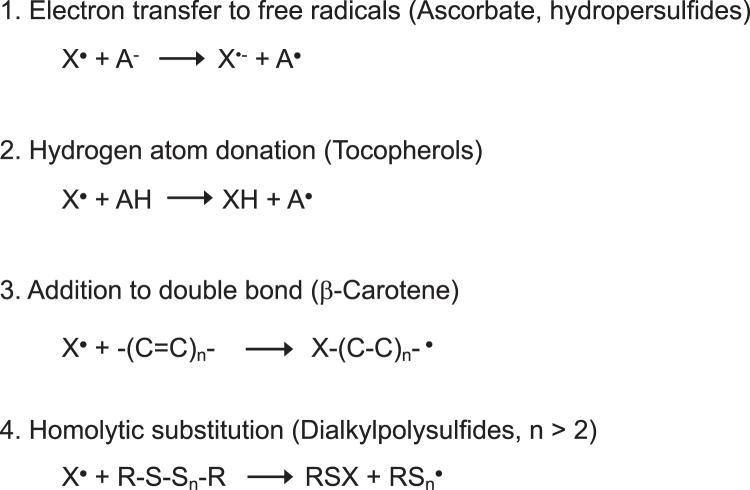
**Four distinct mechanisms of free radical scavenging by antioxidants.** Antioxidants scavenge free radicals, X^•^, by multiple mechanisms: (1) electron transfer, (2) hydrogen atom donation, (3) addition to the double bond, and (4) homolytic substitution. The representative antioxidants for each mechanism are shown in *parentheses*.

Importantly, Chauvin et al. showed that tetrasulfide scavenged peroxyl radicals by the bimolecular homolytic substitution SH2 reaction. This is a characteristic mechanism not seen for other antioxidants. Ascorbate, α-tocopherol, and β-carotene, the three, major, physiological radical scavenging antioxidants, scavenge peroxyl radicals primarily by electron transfer, hydrogen atom donation, and addition to the double bond, respectively (Burton and Ingold, 1984; Takashima et al., 2012) (Fig. 2).

They showed that the stability of the perthiyl radical produced by the homolytic substitution reaction of peroxyl radicals at S2 of the tetrasulfide accounted for the antioxidant activity of tetrasulfide (Chauvin et al., 2019). In other words, the lipid peroxidation chain reaction is not terminated if the homolytic substitution reaction yields the thiyl radical, which (as mentioned above) is not stable, but may attack lipids to continue chain oxidation (Kunath et al., 2020; Trujillo et al., 2016).

An autocatalytic cycle has been proposed recently, in which GSSH reduces radicals to form perthiyl radicals recombining to form GSH tetrasulfide (Reaction 7), which is then reduced by GSH to regenerate GSSH (Reaction 8) (Barayeu et al., 2023).

(7) $$X^{∙}+GSSH\to XH+GSS^{∙}\to GSSSSG$$

(8) GSSSSG+GSH→GSSH+GSSSG

Recently, it was found that disodium disulfide, trisulfide, and tetrasulfide acted as potent peroxyl radical scavengers, the rate constants for scavenging peroxyl radicals being 3.5 × 10^5^, 4.0 × 10^5^, and 6.0 × 10^5^ *M*^−1^·s^−1^ in PBS, pH 7.4, at 37°C, respectively (Kaneko et al., 2022). Furthermore, these sulfides inhibited human plasma lipid peroxidation efficiently at 37°C, the efficacy increasing with the catenation number. Disodium tetrasulfide was 1.5 times as reactive as trolox toward the peroxyl radical.

**FIG. 3.**
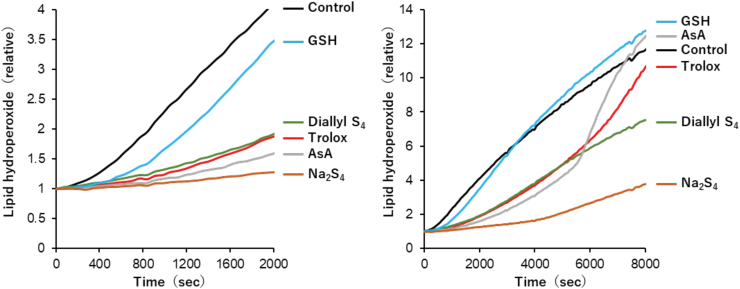
**Antioxidant effects of GSH, AsA, Trolox, Na_2_S_4_, and Diallyl S_4_ against free radical-mediated lipid peroxidation of human plasma (Kaneko et al., 2022).** Human plasma was oxidized by AAPH at 37°C in PBS, pH 7.4, and the formation of lipid hydroperoxides was followed for (*left*) 2000 s and (*right*) 8000 s by using DPPP, which reacts with lipid hydroperoxides specifically to produce fluorescent DPPP oxide. The initial concentrations of plasma, AAPH, and DPPP were 10%, 20 m*M*, and 25 μ*M*, respectively, and that of each antioxidant was 60 μ*M*. AAPH, 2,2′-azobis(2-amidinopropane) dihydrochloride; AsA, ascorbic acid; Diallyl S_4_, diallyl tetrasulfide; DPPP, diphenyl-1-pyrenylphosphine; GSH, glutathione; Na_2_S_4_, disodium tetrasulfide. Color images are available online.

The antioxidant effects against plasma lipid peroxidation induced by free radicals decreased in the order of disodium tetrasulfide > ascorbate > trolox and diallyl tetrasulfide (Fig. 3). It is noteworthy that despite sulfur and oxygen being homologous group 16 elements in the periodic table, hydroperoxide (ROOH) and hydropersulfide (RSSH) exert opposite effects, that is, oxidant and antioxidant effects, respectively.

## Perspectives

**FIG. 4.**
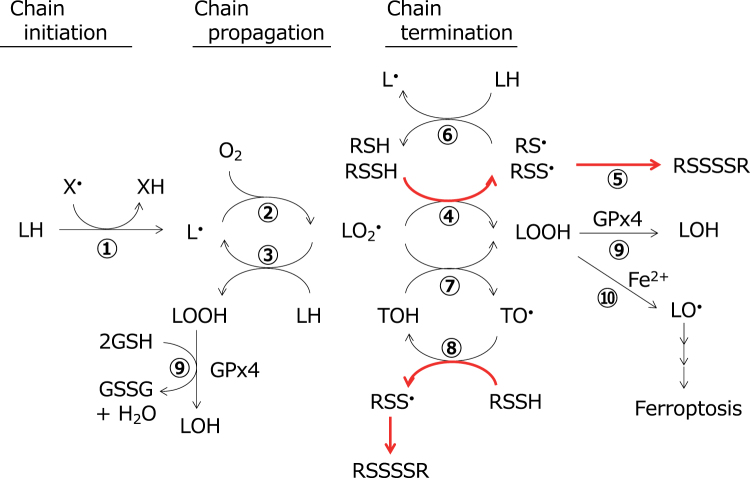
**Inhibition of free radical-mediated lipid peroxidation by thiol, hydropersulfide, and tocopherol.** Lipid peroxidation is initiated by the attack of free radicals (X^•^), including hydroxyl, alkoxyl, peroxyl, nitrogen dioxide, and carbonate anion radicals, on PUFA (LH) to produce the lipid radical (L^•^; Reaction 1), which reacts with oxygen to produce the lipid peroxyl radical (LO_2_^•^; Reaction 2). In the absence of an antioxidant, the lipid peroxyl radical abstracts hydrogen from PUFA (Equation 3) and propagates a chain reaction to produce lipid hydroperoxides (LOOHs). Lipid peroxyl radicals act as chain-carrying species independent of chain-initiating radicals. RSSHs scavenge the lipid peroxyl radical to break chain propagation (Reaction 4). The perthiyl radical (RSS^•^) formed concomitantly is stable and does not propagate chain oxidation, but instead gives a stable product such as tetrasulfide (RSSSSR; Reaction 5). On the other hand, thiol compounds (RSH) such as GSH are much less reactive toward peroxyl radicals and furthermore the resulting thiyl radical (RS^•^) is reactive enough to attack PUFA to initiate chain oxidation (Reaction 6). Therefore, scavenging of the lipid peroxyl radical by thiol compounds does not always result in breaking chain propagation, but it mediates chain transfer. Tocopherol (TOH, vitamin E), the most potent lipophilic antioxidant, scavenges the lipid peroxyl radical (Reaction 7), and the tocopheroxyl radical (TO^•^) produced is reduced by physiological reductants, including vitamin C, ubiquinol, and tocopheryl hydroquinone, to regenerate tocopherol. Interestingly, hydropersulfide is also capable of reducing the tocopheroxyl radical to regenerate tocopherol (Reaction 8). Hydropersulfides act as more potent radical scavenging antioxidants than thiol compounds because of higher reactivity toward peroxyl radicals and higher stability of perthiyl radicals than thiyl radicals. Lipid hydroperoxides (LOOHs) are reduced by the enzyme, GPx4, to the corresponding lipid hydroxides (LOHs) (Reaction 9). Lipid hydroperoxides are decomposed by the ferrous ion to produce lipid alkoxyl radicals, leading to ferroptosis (Reaction 10). GPx, glutathione peroxidase; PUFA, polyunsaturated fatty acid; RSSH, hydropersulfide. Color images are available online.

As described above, hydropersulfides and hydropolysulfides are chemically reactive enough to act as potent radical scavenging antioxidants, and furthermore, the resulting perthiyl and polythiyl radicals are stable, suggesting that they have high potency to inhibit deleterious oxidative events such as lipid peroxidation, as outlined in Figure 4, and that the free radical-mediated antioxidant mechanism may also be important in addition to two-electron mechanisms.

The antioxidant effects are determined by multiple factors, the local concentration of the antioxidant being one of such factors. It was reported that 50–100 μ*M* of per- and polysulfides was detected in mammalian cells, tissues, and plasma (Ida et al., 2014), which is similar to the level of vitamin C in human plasma. However, the results of free radical-induced plasma lipid peroxidation reported from several laboratories showed that lipid peroxidation was inhibited completely by a combination of vitamins E and C, but that oxidation proceeded immediately after vitamin C was consumed (Frei et al., 1988; Itoh et al., 2007), implying that the levels of hydropersulfides and hydropolysulfides in human plasma are much lower than that of vitamin C.

To assess the physiological significance of persulfides and polysulfides as free radical scavenging antioxidants, it is essential to measure their physiological concentrations in human fluids, cells, and tissues. The synergistic interaction between vitamins C and E against lipid peroxidation has been confirmed and whether or not such interaction of hydropersulfides and hydropolysulfides with other physiological antioxidants is significant should be elucidated in future studies.

Many studies observed increased levels of oxidized products derived from vitamin E in the samples of disease patients compared with healthy subjects (Niki and Noguchi, 2021; Torquato et al., 2019). Interestingly, the molar ratio of plasma α-tocopherol quinone to α-tocopherol in atherosclerosis patients was significantly higher than those in healthy subjects.

Furthermore, the molar ratios of nitrated products to parent compounds in healthy human plasma decreased in the order of 5-nitro-γ-tocopherol/γ-tocopherol (10^−2^ mol/mol), 3-nitrotyrosine/tyrosine (10^−5^), nitro-oleic acid/oleic acid (10^−5^), and 8-nitroguanine/guanine (10^−6^), implying that tocopherols act as nitrogen dioxide radical scavenging antioxidants (Niki and Noguchi, 2021). It is important to confirm if hydropersulfides and hydropolysulfides suppress production of free radical-mediated oxidation products from the substrates and antioxidants in cells and *in vivo* under well-defined oxidative stress conditions.

Collectively, it is conceivable that hydropersulfides and hydropolysulfides act as free radical scavenging antioxidants against detrimental oxidation *in vivo*, but the physiological significance remains to be established.

## Abbreviations Used

α-TOH: α-tocopherol
AAPH: 2,2′-azobis(2-amidinopropane) dihydrochloride
AsA: ascorbic acid
CySH: cysteine
Diallyl S_4_: diallyl tetrasulfide
DMPO: dimethyl-1-pyrroline-N-oxide
DNA: deoxyribonucleic acid
DPPP: diphenyl-1-pyrenylphosphine
ESR: electron spin resonance
GPx: glutathione peroxidase
GSH: glutathione
H(p)ETE: hydro(pero)xyeicosatetraenoate
H(p)ODE: hydro(pero)xy-octadecadienoate
LDL: low-density lipoprotein
Mito-DEPMPO: mitochondria-targeted 5-diethoxyphosphoryl-5-methyl-1-pyrroline N-oxide
n.a.: not available
Na_2_S_4_: disodium tetrasulfide
NAFLD: nonalcoholic fatty liver disease
PBD-BODIPY: (T-4)-[3,5-dimethyl-2-[[5-(4-phenyl-1,3-butadienyl)-2H-pyrrol-2-ylidene]methyl]-1H-pyrrolato-N^1^,N^2^]difluoro-boron
POBN: α-(4-pyridyl-1-oxide)-*N*-*tert*-butylnitrone
PUFA: polyunsaturated fatty acid
RS^•^: thiyl radical
RSSH: hydropersulfide
SH2: bimolecular homolytic substitution reaction
TG: triacylglycerol
THF: tetrahydrofuran
